# Extracorporeal photopheresis: A comprehensive review from a transfusion medicine perspective

**DOI:** 10.1016/j.htct.2026.106512

**Published:** 2026-09-04

**Authors:** Suvetha Rajendran, Harsha Unni, Mohandoss Murugesan, Anju R. Kurup, Sangeetha K. Nayanar

**Affiliations:** a Malabar Cancer Centre, Postgraduate Institute of Oncology Sciences and Research, Thalassery, Kerala, India; b Transfusion Medicine, Malabar Cancer Centre, Postgraduate Institute of Oncology Sciences and Research, Thalassery, Kerala, India; c Oncopathology, Malabar Cancer Centre, Postgraduate Institute of Oncology Sciences and Research, Thalassery, Kerala, India

**Keywords:** Extracorporeal photopheresis, 8-methoxypsoralen, Ultraviolet A, Immunomodulation, Leukapheresis

## Abstract

Extracorporeal photopheresis is a unique immunomodulatory therapy that involves the ex vivo treatment of leukocytes with 8-methoxypsoralen and ultraviolet A light, followed by reinfusion into the patient. Initially developed for cutaneous T-cell lymphoma, extracorporeal photopheresis has gained recognition for its efficacy in treating various immune-mediated conditions such as graft-versus-host disease, systemic sclerosis, solid organ transplant rejection, and inflammatory bowel diseases. This review outlines the historical evolution, pharmacology of psoralens, mechanisms of action, clinical applications, equipment types, quality control methods and practical aspects for initiating extracorporeal photopheresis. The therapy's core mechanism, inducing apoptosis and modulating dendritic cell function, promotes immune tolerance and regulatory T-cell expansion. Different inline and offline systems are employed, each with distinct operational and safety considerations. Current evidence supports extracorporeal photopheresis in steroid-refractory graft-versus-host disease and cutaneous T-cell lymphoma, although its role in visceral and long-term disease remains underexplored. Quality control methods such as proliferation assays, flow cytometry, and surrogate markers are discussed in detail. Despite its promise, further randomized trials are necessary to clarify standardized protocols and long-term outcomes.

## Introduction

Extracorporeal photopheresis (ECP) is an immunomodulatory therapy that selectively targets T lymphocytes through the ex vivo combination of psoralen and ultraviolet A (UVA) light, followed by reinfusion of the treated leukocytes [1,2]. It is increasingly recognized for its potential in managing various immune-mediated diseases, including transplant rejection, cutaneous T-cell lymphoma (CTCL), graft-versus-host disease (GvHD), systemic sclerosis, and other autoimmune conditions.

### Brief history

The origins of ECP are rooted in PUVA therapy, a recognized treatment modality for various dermatologic conditions. PUVA combines psoralen, a photosensitizing compound, with UVA exposure. This concept dates back to ancient heliotherapy practices. As early as 1500 BC, Egyptians used plant extracts containing psoralens a furocoumarin compound, notably from *Ammi majus L.*, in combination with sunlight to manage vitiligo. The traditional method involved ingesting a boiled extract from *A. majus*, followed by sun exposure. This is considered the earliest form of PUVA therapy [3].

The period between 1974 and 1988 is often referred to as the era of photochemotherapy. During this time PUVA gained prominence for treating severe psoriasis, mycosis fungoides, and more than 16 other dermatological diseases [4,5]. In 1974, Edelson et al. reported the therapeutic use of leukapheresis in Sézary syndrome. Subsequently, in 1979, Gilchrest documented PUVA therapy’s effectiveness as a palliative treatment for cutaneous T-cell lymphoma (CTCL) [6,7]. Building on these findings, Edelson and his team pioneered the combination of PUVA with leukapheresis, coining the method that would evolve into ECP. Remarkably, two of the first five Phase I leukemic CTCL patients treated achieved remission [2].

### Pharmacology of psoralens

Three psoralens are commonly utilized in PUVA therapy: Methoxsalen (8-methoxypsoralen - 8-MOP), 5-methoxypsoralen and Trioxsalen [8]. Among these, 8-MOP is the most widely used and the only psoralen approved in the United States, owing to its superior gastrointestinal absorption; the others are used predominantly in Europe and other regions. After oral administration, psoralens are rapidly absorbed but undergo first-pass metabolism in the liver, resulting in significant inter-individual variability in plasma concentrations. Approximately 75%–80% of the drug binds to albumin and is distributed throughout the body [9]. In the absence of UVA irradiation, psoralens undergo rapid metabolism and are subsequently excreted in the urine. The serum t_1/2_ of 8-MOP is one hour, but the skin remains sensitive to light for 8–12 hours.

Before UVA exposure, psoralens intercalate into DNA. Upon activation by UVA light (wavelength 320–400 nm), two key types of reactions occur:

• Type I reactions: Oxygen-independent DNA adduct formation.
• Type II reactions: Oxygen-dependent oxidative damage.

These events result in DNA crosslinking, leading to inhibition of replication and the induction of apoptosis. Psoralens also affect mitochondrial function, RNA, protein synthesis, and activate caspases 3 and 9, amplifying apoptotic pathways [10]. Initially, sunlight was the only available UVA source. Between 1974 and 1988, fluorescent phototherapy bulbs became widely adopted to provide controlled, uniform UVA irradiation, with energy doses now measured in joules. Interestingly, UVB radiation also contributes to psoralen activation [11]. Beyond inducing apoptosis, PUVA exerts immunomodulatory effects by altering cytokine and cytokine receptor expression, reducing adhesion molecule expression, and modifying the function of antigen-presenting cells (APCs), such as dendritic cells (DCs) and macrophages [12,13].

### Mechanism of action

ECP is an immunomodulatory therapy effective in conditions such as Sézary syndrome, GvHD, autoimmune diseases, and solid organ transplant rejection. Its therapeutic effects are primarily attributed to three sequential steps:

1. **Leukapheresis** – Peripheral blood mononuclear cells (PBMCs) are collected. During this process, monocytes initiate activation and differentiate toward dendritic cell (DC) lineages.
2. **Photoactivation** – The harvested PBMCs are treated with a photosensitizer (e.g. 8-MOP) and exposed to UVA light, which triggers lymphocyte apoptosis and promotes the maturation of antigen-presenting cells.
3. **Reinfusion** – The treated, apoptotic cells are reinfused into the patient. These cells interact with antigen-presenting DCs, promoting immune tolerance by expanding regulatory T cells (Tregs) and dampening pathogenic immune responses.

ECP exerts its effects through a unique immunoregulatory cascade: initiating apoptosis, modulating DCs, and promoting regulatory T-cell responses. While its mechanism is broadly conserved across conditions, disease-specific pathways influence clinical outcomes.

### Extracorporeal photopheresis in graft-versus-host disease

GvHD is primarily a complication of allogeneic hematopoietic stem cell transplantation, where donor T cells target host tissues. Rarely, this complication may occur after solid organ transplantation, especially with lymphoid-rich organs such as the liver and small intestine [14,15]. Unlike allogeneic hematopoietic stem cell transplantation, solid organ transplantation does not routinely involve prophylaxis against GvHD [16].

ECP has shown promise in steroid-refractory GvHD by modulating the immune response without broad immunosuppression. Mechanistically, ECP enhances Treg expansion, alters cytokine profiles, and supports tolerogenic DC maturation [17]. Notably, DCs and not T cells are central to Treg induction post-ECP. Depletion of CD11c⁺ DCs (but not CD3⁺ T cells) nullifies the suppressive effects. Following reinfusion, while most cells undergo apoptosis, DCs survive for up to 72–96 hours, facilitating antigen presentation in a tolerogenic manner [18]. This delayed apoptosis allows damaged, host-antigen-loaded DCs to present antigens in a tolerogenic manner, promoting Treg rather than effector T-cell responses.

### Extracorporeal photopheresis in cutaneous T cell lymphoma

CTCL, especially its leukemic variant Sézary syndrome, was the first indication for which ECP was developed [19]. It remains the only selective immunotherapy for cancer approved by the Food and Drugs administration (FDA) of the USA. The mechanism involves apoptosis of malignant lymphocytes and their subsequent engulfment by monocyte-derived DCs, initiating adaptive immunity. These DCs mature during the process, upregulating MHC I and II molecules, and present tumor antigens to CD4⁺ and CD8⁺ T cells in lymphoid organs [18,20]. This leads to an anti-tumor Th1 immune response and a beneficial shift in the Th1/Th2 cytokine balance, correlating with clinical improvement [21].

### Extracorporeal photopheresis in allograft rejection and Crohn’s disease

In both solid organ transplant rejection and Crohn’s disease, ECP mitigates immune overactivity through shared mechanisms: apoptosis induction, Treg activation, and cytokine modulation [22]. It suppresses allogeneic reactive T cells and reduces pro-inflammatory cytokines like interleukin (IL)-6 and tumour necrosis factor-alpha.

In chronic rejection syndromes (e.g., bronchiolitis obliterans syndrome), ECP also inhibits transforming growth factor-beta (TGF-β)-mediated fibrosis, offering non–T cell-dependent benefits [23., 24., 25.]. In Crohn’s disease, where CD8⁺ Treg dysfunction is implicated, ECP restores immune regulation without compromising host defenses [26,27]. ECP-treated apoptotic cells also drive IL-10 and TGF-β production by monocytes, promoting a tolerogenic cytokine milieu and reducing intestinal inflammation [28].

### Extracorporeal photopheresis in systemic sclerosis and dermatological diseases

Systemic sclerosis encompasses localized and systemic fibrosing disorders marked by immune dysregulation and collagen accumulation [29]. ECP therapy has been shown to reduce IL-17 levels and enhance anti-inflammatory cytokines, correlating with a decrease in Th17 cells and increased functional Tregs.

Clinically and supported by imaging findings such as high-frequency ultrasound, ECP improves skin elasticity, reduces dermal thickness, and diminishes inflammatory edema [30]. Its immunological effects mimic peripheral tolerance, driven by immature DCs and apoptotic cells that suppress autoreactive lymphocytes [31].

Beyond systemic sclerosis, ECP has also been beneficial in immune-mediated dermatological diseases like atopic dermatitis, lichen planus, and autoimmune bullous disorders, by restoring immune homeostasis through T-cell modulation.

### Equipment used for extracorporeal photopheresis

ECP can be performed using two primary methods: the inline (closed) system and the offline (open) system. In the offline system, leukocyte collection and UVA irradiation are carried out using separate devices. This setup provides flexibility but requires additional manual steps, increasing the risk of contamination. Conversely, the inline system, originally conceptualized by Edelson et al., integrates all steps within a closed, sterile circuit: leukapheresis, photoactivation with 8-MOP, and reinfusion. This method minimizes environmental exposure and contamination risk and is the only FDA-approved system for ECP [32]. Reagents are introduced through sterile connections using bacterial filters, sterile connectors, or under a laminar flow hood. A comparison between the inline and offline systems for ECP is presented in Table 1, and a comparison between the two available inline systems for ECP is provided in Table 2.

**Table 1:** Comparison of inline and offline extracorporeal photopheresis systems.

Inline system	Offline system
Integrates cell collection, UVA irradiation & re-infusion of treated cells within a single closed system	• MNCs are collected using a cell separator • Then, a separate device treats collected cells with 8-MOP and UVA irradiation before manual reinfusion
Advantages:• shorter procedure time• decreased risk of intraprocedural contamination• lower likelihood of improper infusion• single/double needle access• less hands-on time for the operator	Advantages: • flexibility in processing different blood volumes • ability to use different therapeutic protocols on the same cell separator • using a sterile connecting device - closed system
Disadvantages:• higher disposable costs• exclusive use for ECP	Disadvantages: • risk of mix-ups during reinfusion • lack of a single needle option • need for upfront validation of the entire system
Currently available equipment:• Amicus Blue ECP system (Fresenius Kabi, Lake Zurich, IL)• Therakos CELLEX (Mallinckrodt Pharmaceuticals, previously Therakos Inc., West Chester, PA, USA)	Cell separator (standard apheresis device): • SpectraOptia (TerumoBCT) • Com.Tec (FreseniusKabi) • AMICUS (Fenwal) • MCSplus (Haemonetics)
	Drug photoactivation systems• MACOGENICG2 (Macopharma)• UVAPITsystem (MedTechSolutions)• Lumilight (Pelham)

UVA: psoralen and ultraviolet A; MNC: Mononuclear cells; 8-MOP: 8-methoxypsoralen; ECP: Extracorporeal photopheresis.

**Table 2:** Comparison between two inline extracorporeal photopheresis systems.

Parameter	THERAKOS™ CELLEX™	Amicus Blue ECP System
System Type	Inline, closed system	Inline, closed system
Centrifugation Mode	Intermittent flow (DN); continuous (SN)	Continuous centrifugation
Photoactivation Device	Integrated with UVADEX™ automated delivery	Phelix photoactivation module
Needle Configuration	SN and DN, interchangeable	SN and DN supported using one kit
Buffy Coat Handling	Continuous collection with intermittent fluid return	Managed by Amicus separator and Phelix device
UVA Dosage Monitoring	Preset automated exposure. Custom photoactivation time; UVA Light: 1.2 J/cm^2^	Real-time monitoring of UVA dose, temperature, and agitation. UVA Light:1.5 J/cm^2^
Hematocrit Requirement	Minimum 25%	Minimum 15%
Extracorporeal Volume	Approx. 280 mL (DN, HSCT 40%)	Adjustable; supports low total blood volume/HSCT patients (Low 163 mL) [37]
Fluid Management	Automated; red blood cell priming as needed	Real-time fluid balance tracking; custom priming supported. 170 mL saline added during the process
8-MOP	20 µg/mL, 0.017 mL × collected volume	Operator 20 µg/mL (3.4 mL)
System Integration	Fully integrated, closed loop	Integrated Amicus + Phelix with bidirectional communication
Processing Volume	1.5 L	Flexibility to process 500–4000 mL of whole blood
Anticoagulant	Heparin (5000–15,000 IU/500 mL of 0.9% NaCl solution) OR ACD-A between 10:1 and 16:1	ACD A ratio: 12:1 to 16:1
Food and Drug administration Approval	Yes	Yes (Amicus + Phelix system as a combined setup)
Key Advantage	Automated reinfusion, low extracorporeal volume, interchangeable modes	Real-time process monitoring, flexible processing volumes

UVA: psoralen and ultraviolet A; MNC: Mononuclear cells; 8-MOP: 8-methoxypsoralen; ECP: Extracorporeal photopheresis; HSCT: hematopoietic stem cell transplantation; (ACD-A: Anticoagulant Citrate Dextrose Solution, Formula A; SN: Single-needle; DN: Double-needle.

### Technical aspects of extracorporeal photopheresis

The administration of ECP typically follows a structured schedule tailored to the underlying indication and patient response. A standard treatment cycle comprises two consecutive ECP procedures, usually performed on successive days.

• For most indications, one cycle is administered every 2 to 4 weeks.
• In specific conditions such as organ transplant rejection, treatment frequency may be increased to weekly cycles during the initial phase.
• A treatment round generally consists of four consecutive cycles, spread over several weeks.
• The total duration of therapy is typically planned for up to six months, but may be continued until a satisfactory clinical response is achieved.

Treatment intervals and duration may be adjusted based on disease severity, therapeutic goals (e.g., immunomodulation vs. tumor control), and individual patient tolerance. Early tapering or extension of cycles may be guided by clinical improvement, laboratory markers, or imaging findings.

### American Society for Apheresis guidelines for extracorporeal photopheresis

The indications for ECP according to the American Society for Apheresis (ASFA) 2023 guidelines are outlined in Table 3.

**Table 3:** Guidelines for extracorporeal photopheresis as per American Society for Apheresis 2023 [33].

No.	Disease	Indication	Category	Grade	Technical Notes
1.	Atopic Dermatitis		III	2B	One cycle every 2 weeks for 12 weeks. Tapered to every 3–4 weeks, and every 6–12 weeks before stopping.
**2.**	Cutaneous T-cell lymphoma	Erythrodermic mycosis fungoides / Sézary syndrome	I	1B	One cycle – every 2 to 4 weeks After maximal response – tapered to one cycle every 6 to 12 weeks. Stopped if no relapses occur.
		Non-erythrodermic mycosis fungoides	III	2B	
**3.**	Graft-versus-host disease	Acute	II	1B	2 to 3 treatments weekly until response obtained (minimum 8 weeks)
		Chronic	II	1B	One cycle weekly or every other week for 3 months. After response tapered to one cycle/month until maximum clinical benefit is achieved
**4.**	Inflammatory bowel disease	Ulcerative colitis Crohn's disease	III	2C	ECP was given as follows: • Twice weekly for 4 weeks • Twice weekly every other week for 7 weeks • 2 treatments every 2 weeks for 24 weeks
**5.**	Nephrogenic systemic fibrosis		III	2C	Various schedules ranging from 2 on successive days every 2 to 4 weeks up to 5 procedures every other day (cycle) with increasing number of weeks between cycles (1 to 4) with four cycles composing a round.
**6.**	Pemphigus vulgaris	Severe	III	2C	One cycle every 2 or 4 weeks. Continued until clinical response is obtained.
**7.**	Psoriasis	Disseminated pustular	III	2B	One cycle/week for 4 months and then tapered.
**8.**	Systemic sclerosis		III	2A	One cycle per week every 4 to 6 weeks for 6 to 12 months
**9.**	Transplantation, heart	Cellular rejection Recurrent rejection Rejection prophylaxis	II II II	1B 1B 2A	One cycle every week and then tapered Refractory cases: weekly to 6 weeks over 16-to-18-month period.
**10.**	Transplantation, liver	Antibody mediated rejection Immune suppression withdrawal Desensitization ABOi	III III II	2B 2B 2C	One cycle weekly or every 2–8 weeks for several months until liver function improves.
**11.**	Transplantation, lung	Chronic lung allograft dysfunction Bronchiolitis obliterans syndrome	II	1C 1C	Treatment cycles vary between 6 and 24 depending upon the clinical response.

### Quality control (QC) of extracorporeal photopheresis product

The offline ECP system offers a unique advantage in facilitating quality control of the final therapeutic product. Since leukocyte collection and UVA irradiation are performed as discrete steps, this setup allows for the sampling and in vitro validation of treated cells prior to reinfusion. This procedural separation enhances the ability to monitor product consistency, assess cellular viability, and verify the intended biological effects of photoactivation. Various assays can be employed for QC purposes, as summarized in Table 4.

**Table 4:** Comparison of quality control (QC) assays in extracorporeal photopheresis.

QC Method	Principle	Key Features	Advantages	Limitations
Tritiated Thymidine Proliferation Assay	Measures lymphocyte DNA synthesis via ³H-thymidine incorporation after stimulation	Radioactive assay; requires β-scintillation counter	Highly sensitive; established gold standard for proliferation inhibition	Requires radioactive materials; strict regulations; time-consuming (∼5 days)
CFSE-Based Flow Cytometry	Tracks fluorescence dilution with each cell division	Uses CFSE dye; multiparametric flow cytometry	Non-radioactive; precise; allows viability & surface marker analysis	Requires flow cytometry expertise; 3-day culture; reagent validation needed
Annexin V/Propidium Iodide Assay	Quantifies early and late apoptosis via membrane integrity changes	Flow cytometry; Annexin V⁺/PI⁺ gating	Quick (24 hours); simple protocol; direct measure of apoptosis	Does not measure proliferation; limited functional insight
T-cell Activation Markers (e.g., CD71, Ki-67)	Detects suppression of activation markers post-ECP	Flow cytometric surface staining	Rapid; reliable; no cell culture or radioactive handling required	Surrogate measure; may not fully reflect functional proliferation inhibition

**Tritiated thymidine proliferation assay:** This assay is based on the principle that 8-MOP, upon UVA activation, covalently binds to DNA, inhibits replication, and induces apoptosis in lymphocytes, typically within 72 hours post-irradiation. The assay evaluates the disappearance of proliferative capacity in lymphocytes pre- and post-ECP. Cells are cultured in RPMI medium and stimulated with phytohemagglutinin (PHA) in 50% of the wells. ³H-thymidine is added during the final 18 hours of a three-day incubation period to measure DNA synthesis, which represents cell proliferation. Incorporation is quantified using a β-scintillation counter, providing a sensitive measure of proliferation inhibition. However, this method involves the use of radioactive material, necessitating stringent safety and regulatory oversight [34].

**Carboxyfluorescein succinimidyl ester (CFSE) based flow cytometry**: An effective, non-radioactive alternative is the use of CFSE for tracking lymphocyte proliferation via flow cytometry [35]. CFSE passively diffuses into cells and binds to intracellular proteins; the fluorescence intensity is decreased with each cell division. Cells are initially labelled with CFSE, with two aliquots (pre- and post-ECP) being prepared for each sample. One aliquot is stimulated with PHA, and both are incubated for 72 hours. Flow cytometric analysis focuses on CFSE⁺/7-AAD⁻/CD3⁺ events. The percentage of proliferating cells and the inhibition rate are then calculated. This assay is precise at the single-cell level, does not involve hazardous materials, and allows for multicolor immunophenotyping and viability assessment within a single tube. Faivre et al. validated the method per ISO 15189:2007 standards, showing strong concordance with the thymidine assay, and demonstrating its robustness, reproducibility, and suitability for routine QC [34].

**Annexin V/Propidium iodide apoptosis assay:** Taverna et al. employed Annexin V/PI double staining to quantify the extent of apoptosis induced in ECP-treated mononuclear cells [36]. Cells are gated using forward and side scatter, and categorized as follows:

• **Viable cells**: Annexin V⁻/PI⁻.
• **Early apoptosis**: Annexin V⁺/PI⁻.
• **Late apoptosis**: Annexin V⁺/PI⁺.

The net apoptosis (ΔApoptosis) is determined by comparing the sum of early and late apoptotic populations in pre- and post-ECP samples. This method is quicker (within 24 hours) and simpler than proliferation assays, though it does not provide functional information about the residual proliferation capacity [36].

**T-cell activation marker analysis:** To streamline QC and reduce assay time, Schwab et al. evaluated surface expression of activation markers, such as CD71, Ki-67, CD25, and CD69, on T cells after ECP [37]. Of these markers, CD71 demonstrated the most consistent and rapid upregulation within 6–8 hours post-activation, an expression that was nearly abolished by ECP treatment. While CD71 lacks a significant logistical time advantage over CFSE in clinical workflows, it represents a reliable and technically simple surrogate marker to assess the inhibition of T-cell proliferation, which is particularly useful when other T-cell-suppressive agents are not in use.

### Limitations and gaps

Despite its established role in several immune-mediated conditions, ECP has important limitations. The mechanism of action remains incompletely understood, particularly in relation to visceral organ involvement, limiting its broader clinical application. Most evidence is derived from cutaneous conditions like CTCL and skin-dominant GvHD, with limited data on efficacy in internal organ diseases such as pulmonary or gastrointestinal GvHD and systemic sclerosis with fibrosis.

There is a lack of standardized treatment protocols, leading to variability in the clinical practice across centers. Additionally, the absence of large, randomized controlled trials weakens the strength of current evidence, which is largely based on case series and expert opinion. Long-term outcome data are also sparse, and reliable biomarkers to predict or monitor treatment response remain elusive. These gaps highlight the need for mechanistic research, controlled studies, and biomarker development to optimize and expand the use of ECP.

### Practical steps for initiating extracorporeal photopheresis in patient care

Implementing ECP requires a structured, multidisciplinary approach encompassing pre-procedural preparation, intraprocedural monitoring, and post-procedural care to ensure safety and optimize therapeutic benefit. The first step is careful patient selection based on clearly defined indications. According to the ASFA, ECP is recommended as a first- or second-line therapy for conditions such as steroid-refractory GvHD, cutaneous T-cell lymphoma (CTCL), and selected cases of transplant rejection and autoimmune diseases [33].

Before initiating therapy, a thorough clinical evaluation and laboratory investigations should be performed. These include a complete blood count, liver and renal function tests, assessment of venous access, and evaluation for contraindications to psoralen or UVA exposure. Special consideration is required in patients with photosensitive disorders or hepatic impairment due to the pharmacokinetics of 8-MOP. Informed consent should detail the planned treatment duration, expected frequency, potential side effects, and the necessity of a prolonged follow-up [24].

During the intraprocedural phase, patients are typically treated using an inline system (e.g., Therakos™ CELLEX™ or Amicus™ Blue) which integrates leukapheresis, photoactivation with UVA, and reinfusion. Vascular access via double-needle configuration is preferred for optimal flow rates, though single-needle systems are available for patients with limited access. Anticoagulation is typically achieved with an Anticoagulant Citrate Dextrose Solution, Formula A (ACD-A); monitoring for citrate-related toxicity is essential, especially in pediatric or low-weight patients [32]. The UVA dose is pre-set based on manufacturer recommendations (usually 1.2–1.5 J/cm²), and photoactivation occurs in a sterile environment with precise control of exposure time and temperature.

If oral 8-MOP is used (typically in offline systems), it must be administered two hours prior to UVA exposure, and patients are required to avoid sunlight and wear protective eyewear for 24–48 hours after ingestion. During treatment, continuous monitoring of vital signs and extracorporeal volume is essential to identify and manage any infusion-related adverse events such as hypotension, chills, and nausea [24].

Post-procedurally, patients should be observed for immediate complications and encouraged to maintain hydration. Photosensitivity precautions must be reinforced, particularly following oral psoralen administration. Documentation of key procedural parameters, including total blood volume processed, hematocrit, anticoagulant ratio, and UVA exposure, is critical for tracking treatment response. Long-term evaluation should include periodic clinical assessments tailored to the underlying disease, such as GvHD staging or modified Rodnan Skin Score (mRSS) for systemic sclerosis. Treatment schedules are typically individualized, beginning with cycles every two to four weeks, and adjusted based on clinical response [33].

## Conclusion

ECP is an evolving therapeutic tool in transfusion medicine. It uniquely combines immunologic specificity and systemic tolerability. Nevertheless, gaps remain regarding its precise mechanisms, especially in visceral disease settings, and robust evidence from large controlled studies is needed to solidify protocols, clarify long-term outcomes, and refine its application across diverse patient groups. Continued research into mechanistic pathways, standardized QC assays, predictive biomarkers, and long-term follow-up will be essential for maximizing the therapeutic value and safe integration of ECP into broader clinical practice.

## Permission to reproduce material from other sources

Not required.

## Author contribution statement

Suvetha Rajendran: Conceptualization, Formal analysis, Validation, Writing - Original Draft. Harsha Unni: Conceptualization, Formal analysis, Validation, Writing - Original Draft. Mohandoss Murugesan: Conceptualization, Formal analysis, Validation, Writing - Original Draft, Writing - Review & Editing. Anju R Kurup: Methodology, Supervision. Sangeetha K Nayanar: Methodology, Supervision.

## Declaration of generative AI in scientific writing

During the preparation of this work the authors used ChatGPT for language editing. After using this tool, the authors reviewed and edited the content as needed and take full responsibility for the content of the publication.

## Funding statement

We declare that no financial support for the research, authorship, and/or publication of this article was received.

## Data availability statement

Not applicable.

## Conflicts of interest

The authors declare that they have no competing interests.
